# Conscience clause in brain death care: ethical and legal perspectives of young nurses and midwives in Poland

**DOI:** 10.3389/fmed.2025.1683300

**Published:** 2025-10-13

**Authors:** Justyna Czekajewska, Dariusz Walkowiak, Anna Jelińska, Jan Domaradzki

**Affiliations:** ^1^Department of Social Sciences and Humanities, Poznan University of Medical Sciences, Poznan, Poland; ^2^Department of Organization and Management in Health Care, Poznan University of Medical Sciences, Poznan, Poland; ^3^Department of Pharmaceutical Chemistry, Poznan University of Medical Sciences, Poznan, Poland

**Keywords:** brain death, conscience clause, end-of-life care, ethical and legal dilemmas, medical ethics, midwives, nurses, religiosity

## Abstract

**Background:**

The conscience clause allows healthcare professionals to refuse participation in procedures that conflict with their ethical or religious beliefs. While intended to protect moral autonomy, its application, particularly in the context of brain death and organ donation, raises complex ethical and legal challenges. This study aims to explore the attitudes of young nurses and midwives in Poland toward the conscience clause in relation to brain death, including their ethical concerns, perceived consequences, and views on legal regulations.

**Methods:**

A cross-sectional survey was conducted among 269 master’s students of nursing and midwifery at the Medical University in Poznan, Poland. Participants completed a questionnaire covering ethical dilemmas, legal opinions, and potential consequences related to invoking the conscience clause. Descriptive statistics and logistic regression were used to analyze the data, with significance set at *p* < 0.05.

**Results:**

Most respondents supported both respecting patients’ beliefs (90.3%) and the right to act according to their own conscience (68.4%). However, they expressed concerns about negative social and professional consequences, such as strained relationships (40.2%), job loss (43.9%), or legal action (56.9%). Attitudes were shaped by religiosity and political views. Religious participants were more likely to support conscientious objection in organ retrieval (OR = 2.08). At the same time, right-leaning students were less likely to support invoking the clause when mandated to disconnect a brain-dead patient. Respondents favored structured national regulations (81.8%) over institutional discretion or full abolition of the clause.

**Conclusion:**

Young healthcare professionals report facing significant ethical and legal dilemmas related to brain death, and their views appear to be associated with personal values and societal context. The findings highlight the need for standardized guidelines, legal protections, ethics education, and further qualitative research to support ethically sound and patient-centered practice.

## Background

The conscience clause is a legal provision that permits healthcare professionals to refuse to perform certain medical procedures if these conflict with their ethical or religious beliefs (1). At the same time, it is important to distinguish between the conscience clause and conscientious objection. The conscience clause refers to the formal legal framework that grants healthcare professionals the right to refuse certain procedures within a regulated healthcare system. By contrast, conscientious objection refers to an individual’s moral decision to decline participation in an action perceived as conflicting with their core ethical or religious beliefs, thereby protecting their personal moral integrity (2, 3). While designed to safeguard moral integrity and freedom of conscience, the application of the conscience clause often creates complex ethical and legal dilemmas, particularly in end-of-life care, brain death, and organ transplantation (4). One of the most ethically sensitive situations in clinical practice is the determination of brain death, which functions as both a legal and medical prerequisite for *ex mortuo* organ donation (5). Nurses and midwives, especially those working in intensive care units or serving as transplant coordinators, are frequently involved in caring for such patients (6). These professionals may experience moral distress when their duties conflict with their personal beliefs (7).

Studying the attitudes of nurses and midwives, therefore, provides valuable insight into the ethical and legal dilemmas arising from value conflicts. Unresolved issues may lead to emotional strain, professional burnout, reduced care quality, or even job resignation (8, 9). Gaining such knowledge helps assess whether staff feel able to voice objections and what support they require, while also informing the development of realistic legal procedures that uphold both continuity of care and the rights of healthcare workers.

Brain death is defined as the irreversible cessation of all brain functions, including those of the brainstem, and is legally recognized as death in many countries, including Poland (10–12). In 2019, Poland updated its brain death regulations to align with international standards (13).

According to current guidelines, two medical specialists must confirm the diagnosis: one in anesthesiology, intensive care, or neonatology, and the other in neurology, pediatric neurology, or neurosurgery. The process includes a clinical assessment to confirm lack of responsiveness, absence of brainstem reflexes, and a positive apnea test. Physicians must also exclude confounding factors such as hypothermia, hypotension, metabolic disturbances, or sedative drugs. If clinical uncertainty remains, confirmation requires instrumental tests such as electroencephalography, cerebral angiography, or validated neuroimaging methods (13). Only after brain death is clearly established according to these neurological criteria can the process of post-mortem organ procurement begin.

Despite its clear legal and medical definition, brain death continues to raise ethical concerns among healthcare professionals. These include doubts about the adequacy of diagnostic criteria, decisions regarding withdrawal of life-sustaining treatment, the process of informing families, and participation in organ procurement procedures (14, 15). Such dilemmas are particularly acute for nurses and midwives, who provide direct care to brain-dead patients and maintain close contact with their families (16, 17).

Importantly, these challenges are not limited to Poland. Despite systemic and cultural differences, ethical concerns among healthcare professionals are a global phenomenon. Studies show that 60 to 90% of healthcare workers report encountering ethical issues in clinical practice (18–24). Similar patterns are observed among medical students. Research by Stępień and Tkaczyk found that 59% of students saw worldview-related conflicts as ethically troubling, and 34% viewed futile therapy as morally problematic (25). Likewise, Ścieranka et al. demonstrated that students face dilemmas when asked to perform tasks beyond their competence or when unsure about their responsibilities (26). Although nursing students display a relatively high level of knowledge about brain death (73.9%) and its diagnostic principles (77.1%), this knowledge still varies depending on workplace and education level (15, 26, 27).

The topic of *post-mortem* transplantation also raises multiple ethical and legal concerns (28). Nurses caring for brain-dead patients often raise concerns. They question whether brain death is a sufficient criterion for declaring death, worry about the objectification of donors, and feel uneasy when informing families about death or possible donation (16, 17, 29–31). Some medical personnel also refuse to participate in the withdrawal of life support or artificial feeding, often due to religious or ethical convictions (32, 33). In such cases, they may invoke the conscience clause – a legal provision allowing healthcare professionals to opt out of procedures that conflict with their beliefs (22, 34).

In Poland, the conscience clause is explicitly regulated and applies to certain professional groups, including physicians, nurses, and midwives. For nurses and midwives, this is governed by Article 12 of *the Act on the Professions of Nurse and Midwife* (35). The law allows nurses and midwives to refuse participation in services that conflict with their conscience, but only if they notify their supervisor in advance and record the objection in the patient’s file (35, 36). Refusal is not permitted in emergencies or when a patient’s life or health is at risk. Unlike physicians, nurses and midwives are also legally required to indicate where the service can be accessed, for example, by referring the patient to another professional or facility (35). Physicians are exempt from this obligation due to a 2015 Constitutional Tribunal ruling, which argued that such referrals would make them complicit in acts that violate their conscience (37). Responsibility for ensuring continuity of care in these cases lies with the medical institution’s director.

According to recent research, 87% of religious and 81% of religiously ambivalent healthcare workers believe nurses should have priority access to the conscience clause. Furthermore, 46% of religious and 70% of ambivalent respondents agree that if a service is refused, there should be a legal obligation to refer the patient to another provider (34). These findings underscore the importance of further research on the topic.

In Poland, the number of nurses working in anesthesiology, intensive care, and transplant coordination is steadily increasing, which has a positive impact on organ donation rates (6, 16, 38, 39). According to Poltransplant data from 2022, among 332 hospital coordinators, 113 were nurses, 212 were physicians, and 7 represented other medical professions (40). Given this growing involvement, understanding nurses’ ethical and legal perspectives becomes increasingly important.

In challenging professional situations, 44% of medical staff report being guided primarily by patient welfare, 41% by their own conscience, and only 32% by legal regulations (25). This highlights the importance of healthcare professionals striking a balance between upholding patient rights and adhering to their own personal values.

To date, most studies on the conscience clause have focused on physicians, particularly in the context of abortion (41). The application of this provision by Polish nurses and midwives, especially in the context of brain death, remains under-researched. There is also limited research that incorporates Polish cultural and legal specifics or examines the clause’s impact on team dynamics (4, 42–44).

This study, therefore, represents one of the first attempts to explore these issues among Polish master’s students in nursing and midwifery and fills a significant gap in the literature by examining how they perceive: (1) ethical and legal dilemmas related to the conscience clause in brain death scenarios; (2) concerns about the consequences of invoking the clause; (3) perceptions of legal frameworks and preferred regulatory solutions; (4) sociodemographic correlates of selected legal provisions; and (5) associations between sociodemographic factors and ethical positions in brain death cases.

A deeper understanding of these perspectives can inform institutional protocols, legal protections, and educational programs that balance professional autonomy with patient rights, shaping health policy on ethical conflicts and continuity of care in end-of-life and organ donation contexts.

## Methods

### Study design

This study was part of a larger project on healthcare professionals’ attitudes toward ethical challenges in defining and diagnosing brain death. Its specific aim was to assess how young nurses and midwives view the ethical and legal dilemmas of the conscience clause in this context.

Data were collected with an anonymous paper-based questionnaire completed by master’s students in nursing and midwifery at the Poznan University of Medical Sciences (PUMS).

Based on a literature review, a questionnaire was designed to assess how Polish nursing and midwifery students perceive ethically and legally controversial issues related to the conscience clause in brain death. It also examined concerns about its consequences, relevant legal regulations, and the influence of sociodemographic factors (45).

### Participants and setting

The study recruited master’s students in nursing and midwifery for two main premises. First, after completing a three-year undergraduate program, they already hold professional qualifications and usually begin working in healthcare facilities. Second, their clinical practice often involves terminally ill patients, exposing them to ethical dilemmas and potential moral conflicts.

Eligibility criteria included being a nursing or midwifery student at the PUMS, fluency in Polish, and providing informed consent before completing the questionnaire.

### Research tools

Based on a literature review concerning the conscience clause in the context of brain death and in collaboration with experts in public health, medical sociology, and bioethics, a research questionnaire was developed. Its structure was designed in accordance with the guidelines of the European Statistical System (46). A pilot version of the tool was tested among 115 undergraduate students in midwifery and emergency medical services, resulting in the reformulation of three questions.

The final version of the questionnaire consisted of four sections. The first contained questions regarding the opinions of young nurses and midwives about the conscience clause in relation to brain death. The second section included questions about their concerns related to the conscience clause in the context of brain death. The third part focused on verifying respondents’ opinions about the legal regulations concerning the conscience clause and the best ways to address them. The last section of the questionnaire included questions on respondents’ demographic characteristics.

All questions were closed-ended, with a defined set of clear response options. To ensure clarity and facilitate responses, specialized terminology was avoided. The question design allowed respondents to express their degree of agreement on a five-point Likert scale, from “Strongly disagree” to “Strongly agree,” which enabled participants to express their stance clearly. Most questions also included a neutral option: “I do not know”.

### Data collection

The study was conducted between March and June 2024 among master’s students in nursing and midwifery at PUMS. Using purposive sampling, questionnaires were distributed during regular classes for fourth- and fifth-year full-time students.

Before participation, all students were informed about the purpose of the project and provided written consent. The principal investigator (JC) was present throughout data collection to address questions. Completing the questionnaire took on average 10–15 min.

### Ethical issues

The study was conducted in accordance with the principles outlined in the Declaration of Helsinki (revised in 2000) (47) and received approval from the Bioethics Committee of the Poznan University of Medical Sciences (approval no. KB-07/24, decision dated January 3, 2024). Before participation, all respondents provided informed and voluntary consent to participate in the study, as confirmed in written form.

### Data analysis

Descriptive statistics were used to summarize the sociodemographic characteristics and responses to the Likert scale items, which are presented as frequencies and percentages of the total responses. Logistic regression was employed to examine associations between sociodemographic variables and factors related to students’ attitudes toward the conscience clause in the context of brain death. All statistical analyses were performed using JASP (version 0.19.3), with a significance level set at *p* < 0.05 (48).

## Results

Table 1 presents the socio-demographic characteristics of the participants (*N* = 269). Most respondents were female (97.4%), with a mean age of 23.9 years (range 22–50). Students were almost evenly split between nursing (46.5%) and midwifery (53.5%), and between the fourth (45.7%) and fifth year of study (54.3%).

**Table 1:** Socio-demographic characteristics of the study participants (Poland, 2024).

Characteristics	Total	
	(*N* = 269)	% *N*
Gender		
Female	262	97.4%
Male	5	1.9%
Non-binary	2	0.7%
Faculty		
Nursing	125	46.5%
Midwifery	144	53.5%
Year of the study		
Fourth year	123	45.7%
Fifth year	146	54.3%
Age (in years)		
Range	22–50	
Mean (95%CI)	23.9 (23.5–24.2)	
Standard deviation	3.1	
Median	23	
Participation in a professional internship		
Yes	227	84.4%
No	42	15.6%
The role of religion plays in your life		
High/moderate	79	29.4%
Low/none	190	70.6%
Liberal–conservative orientation (worldview beliefs)		
Strongly/rather liberal	149	55.4%
Centrist	102	37.9%
Rather/strongly conservative	18	6.7%
Political preferences on the left–right spectrum		
Left	135	58.2%
Center	103	38.3%
Right	31	11.5%

A majority (84.4%) had completed a professional internship. Religion played a high or moderate role in the lives of 29.4% of students, while 70.6% reported little or no influence. In terms of worldview, 55.4% identified as liberal, 37.9% as centrist, and 6.7% as conservative. Regarding political preferences, 58.2% identified with left-leaning views, 38.3% positioned themselves at the center, and 11.5% expressed right-leaning preferences.

Table 2 presents the perspectives of young nurses and midwives on the conscience clause in the context of brain death. Most respondents agreed (90.3%) that medical professionals should always respect patients’ beliefs, even if they differ from their own. Views were more divided on whether saving human life is an absolute duty that overrides conscientious objection (59.5% agreed, 18.6% were unsure). Overall, 68.4% supported the right of professionals to act according to their conscience, while most opposed allowing employers to ask about such views during recruitment (53.9%).

**Table 2:** Young nurses’ and midwives’ views on the conscience clause towards brain death (Poland, 2024).

Question	Definitely not *n* (%)	Rather not *n* (%)	I do not know *n* (%)	Rather yes *n* (%)	Definitely yes *n* (%)
Should a medical professional always respect their patients’ beliefs, even if they differ from their own?	7 (2.6)	8 (3)	11 (4.1)	73 (27.1)	170 (63.2)
Is saving human life and health a duty of a medical professional, meaning they cannot refuse to perform a procedure even if it goes against their conscience?	22 (8.2)	37 (13.8)	50 (18.6)	81 (30.1)	79 (29.4)
Should medical professionals have the right to perform their duties in accordance with their own conscience?	11 (4.1)	29 (10.8)	45 (16.7)	70 (26)	114 (42.4)
Should an employer have the right to ask a candidate applying for a position in a medical institution about their personal beliefs regarding the conscience clause in the context of brain death?	64 (23.8)	81 (30.1)	69 (25.7)	35 (13)	20 (7.4)
Should the law require a medical professional who, due to conscientious objection, refuses to perform a medical procedure to indicate another professional who would perform it instead?	34 (12.6)	37 (13.8)	76 (28.3)	63 (23.4)	59 (21.9)
Can personal beliefs about human death be a reason for medical professionals to invoke the conscience clause?	24 (8.9)	37 (13.8)	63 (23.4)	86 (32)	59 (21.9)
If the disconnection of a patient in a state of brain death were mandated, would you like to have the option to invoke the conscience clause?	33 (12.3)	58 (21.6)	78 (29)	63 (23.4)	37 (13.8)
In the case of preparing a deceased donor who has died due to brain death for organ retrieval, can a medical professional invoke a conscientious objection and refuse to perform this procedure?	43 (16)	59 (21.9)	42 (15.6)	79 (29.4)	46 (17.1)
Since the Polish Constitution and international law guarantee the right to freedom of thought, conscience, and religion, should the definition of human death be an individual choice rather than being determined by scientific considerations?	69 (25.7)	62 (23)	64 (23.8)	54 (20.1)	20 (7.4)
Is it important to establish uniform medical regulations worldwide to develop safety standards and prevent abuses, such as treating bodies of brain-dead individuals as incubators or corpses?	4 (1.5)	10 (3.7)	35 (13)	103 (38.3)	117 (43.5)

Responses were mixed on whether objecting professionals should be obliged to indicate a substitute (45.3% agreed, 28.3% undecided) and whether personal beliefs about death could justify objection (53.9% agreed). Attitudes toward disconnection of a brain-dead patient and organ retrieval also revealed division, with only about one-third expressing support for invoking the clause in these cases (37.2%). In the case of organ retrieval from brain-dead donors, 46.5% of respondents agreed that medical professionals should have the right to refuse participation.

Most students rejected the idea that defining death should be a matter of personal belief (48.7% disagreed), while strong support was expressed for globally unified regulations to prevent ethical abuses (81.8%).

Table 3 presents the concerns of young nurses and midwives regarding the conscience clause in the context of brain death. Many respondents expressed apprehension about the potential negative consequences of invoking the clause. The most common concern was strained relationships with colleagues, reported by 40.2%. Similarly, 45.7% feared negative opinions or gossip.

**Table 3:** Young nurses and midwives’ concerns related to the conscience clause in the context of brain death (Poland, 2024).

Concerns related to the conscience clause in the context of brain death	Definitely not *n* (%)	Rather not *n* (%)	I do not know *n* (%)	Rather yes *n* (%)	Definitely yes *n* (%)
Fear that relationships with colleagues will deteriorate and become tense and conflict-ridden	67 (24.9)	81 (30.1)	13 (4.8)	93 (34.6)	15 (5.6)
Fear of not receiving a professional promotion	81 (30.1)	115 (42.8)	16 (5.9)	46 (17.1)	11 (4.1)
Fear that it will result in negative opinions/gossip about me	64 (23.8)	69 (25.7)	13 (4.8)	101 (37.5)	22 (8.2)
Fear that it will lead to the denial of support from colleagues in difficult situations	64 (23.8)	63 (23.4)	14 (5.2)	108 (40.1)	20 (7.4)
Fear of being treated with contempt and disregard by colleagues and patients	65 (24.2)	79 (29.4)	18 (6.7)	88 (32.7)	19 (7.1)
Fear that colleagues will withhold important information	56 (20.8)	90 (33.5)	27 (10)	84 (31.2)	12 (4.5)
Fear of disciplinary proceedings	59 (21.9)	80 (29.7)	18 (6.7)	84 (31.2)	28 (10.4)
Fear of a potential civil lawsuit from the family	39 (14.5)	56 (20.8)	21 (7.8)	116 (43.1)	37 (13.8)
Fear of losing the job	52 (19.3)	75 (27.9)	24 (8.9)	86 (32)	32 (11.9)

Concerns about professional advancement were also noted, although they were less prominent: 59.9% disagreed that conscientious objection would harm promotion opportunities. Still, 47.5% worried that refusal to participate might lead to a lack of support from colleagues, while 39.8% feared being treated with contempt. A smaller group, 35.7% reported concern about colleagues withholding important information. Legal and disciplinary consequences were also noted: 41.6% feared disciplinary proceedings, and 56.9% feared a civil lawsuit from a patient’s family. 43.9% expressed concern that invoking the conscience clause could threaten job security. These findings underscore the perceived professional and social risks associated with conscientious objection in the context of brain death.

Table 4 presents young nurses’ and midwives’ perspectives on legal regulations concerning the conscience clause in the context of brain death. Respondents expressed varied views on how the clause should be regulated in medical practice. A majority (65.4%) supported national agreements developed exclusively by medical experts, whereas 43.5% favoured regulations decided solely by medical professionals. By contrast, 67.3% opposed giving decision-making authority exclusively to hospital management.

**Table 4:** Young nurses and midwives’ views on legal regulations related to the conscience clause in the context of brain death (Poland, 2024).

Regulation approach	Definitely not *n* (%)	Rather not *n* (%)	I do not know *n* (%)	Rather yes *n* (%)	Definitely yes *n* (%)
Through national agreements made exclusively by medical experts					
	2 (0.7)	39 (14.5)	52 (19.3)	113 (42)	63 (23.4)
Through decisions made exclusively by medical professionals					
	25 (9.3)	84 (31.2)	43 (16)	93 (34.6)	24 (8.9)
Through decisions made exclusively by the management of a specific hospital, where an employee invoked the conscience clause					
	76 (28.3)	105 (39)	52 (19.3)	29 (10.8)	7 (2.6)
By establishing uniform criteria for human death worldwide					
	8 (3)	14 (5.2)	34 (12.6)	91 (33.8)	122 (45.4)
By introducing specific ethical and legal regulations defining the scope, conditions, and medical procedures that employees are obliged to follow					
	7 (2.6)	8 (3)	40 (14.9)	97 (36.1)	117 (43.5)
By completely abolishing the conscience clause					
	73 (27.1)	75 (27.9)	72 (26.8)	28 (10.4)	21 (7.8)

The idea of establishing uniform global criteria for defining human death received broad support (79.2%). Similarly, 79.6% agreed that there should be clear ethical and legal regulations specifying when and how healthcare professionals may invoke the conscience clause. Views on abolishing the conscience clause entirely were more divided: 55% disagreed, 26.8% were uncertain, while only 18.2% agreed.

These findings indicate a strong preference for structured national and international guidelines, coupled with clear opposition to hospital-based control and limited support for abolishing the conscience clause.

Table 5 presents the logistic regression results examining associations between sociodemographic factors and young nurses’ and midwives’ preferences regarding legal approaches to regulating the conscience clause in the context of brain death. One significant predictor was the professional group: respondents in midwifery were significantly less likely than those in nursing to support exclusive decision-making by medical professionals (OR = 0.480, 95% CI: 0.274–0.841, *p* < 0.05). Support for introducing specific ethical and legal regulations, defining the scope, conditions, and required medical procedures, was significantly associated with religious beliefs: individuals with religious beliefs were less likely to favour this approach (OR = 0.357, 95% CI: 0.142–0.902, *p* < 0.05).

**Table 5:** Logistic regression parameters for preferences on legal regulations of the conscience clause (Poland, 2024).

Regression parameters	Through decisions made exclusively by medical professionals	Through decisions made solely by the management of a specific hospital, where an employee invoked the conscience clause	By introducing particular ethical and legal regulations defining the scope, conditions, and medical procedures that employees are obliged to follow	By completely abolishing the conscience clause
	OR (95%CI)	OR (95%CI)	OR (95%CI)	OR (95%CI)
Intercept	1.987*** (1.908;4.321)	-	10.500*** (2.463;44.761)	0.227*** (0.196;0.426)
Age				
Midwifery vs. Nursing	0.480* (0.274;0.841)			
Religious vs. nonreligious				0.357* (0.142;0.902)
Left vs. right			0.648 (0.138;3.045)	
Right vs. centre			0.181* (0.040;0.824)	
R^2^ Nagelkerke	0.060		0.108	0.043
*p*-value for Model	0.01		<0.001	0.028

* *p*-value < 0.05; ** *p*-value < 0.01; *** *p*-value < 0.001.

Regarding the complete abolition of the conscience clause, right-leaning individuals were significantly less likely to support this compared to centrists (OR = 0.181, 95% CI: 0.040–0.824, *p* < 0.05). Model fit, as measured by Nagelkerke’s R^2^, varied, with the highest explanatory power found for the model predicting support for ethical and legal regulations (R^2^ = 0.108). All models reached statistical significance, with the strongest association observed in the one predicting support for ethical and legal regulation (*p* < 0.001).

Table 6 presents logistic regression results examining factors associated with the likelihood that young nurses and midwives would invoke the conscience clause in three contexts: organ retrieval from a brain-dead donor, personal beliefs about human death, and mandated disconnection of a brain-dead patient.

**Table 6:** Logistic regression parameters for the conscience clause in organ retrieval and brain death scenarios (Poland, 2024).

Regression parameters	In the case of preparing a deceased donor who has died due to brain death for organ retrieval, can a medical professional invoke a conscientious objection and refuse to perform this procedure?	Can personal beliefs about human death be a reason for medical professionals to invoke the conscience clause?	Would you like to have the option to invoke the conscience clause if the disconnection of a patient in a state of brain death were mandated?
	OR (95%CI)	OR (95%CI)	OR (95%CI)
Intercept	3.000 (0.812; 11.081)	0.986 (0.714; 1.363)	-
Age			
Midwifery vs. nursing			
Religious vs. nonreligious		2.076* (1.123; 3.835)	
Left vs. right	0.339 (0.087; 1.314)		
Right vs. centre	0.200* (0.050; 0.797)		
R^2^ Nagelkerke	0.046	0.035	
*p*-value for model	0.025	0.018	

* *p*-value < 0.05; ** *p*-value < 0.01; *** *p*-value < 0.001.

Religious affiliation was significantly associated with the willingness to invoke the conscience clause in the context of organ retrieval: participants with a religious affiliation were more likely to support conscientious objection (OR = 2.076, 95% CI: 1.123–3.835, *p* = 0.025). Similarly, right-leaning individuals were significantly less likely than centrists to support invoking the clause when required to disconnect a brain-dead patient (OR = 0.200, 95% CI: 0.050–0.797, *p* = 0.018).

The models for organ retrieval and personal beliefs about human death had limited explanatory power, with Nagelkerke R^2^ values of 0.046 and 0.035, respectively. However, both models reached statistical significance, indicating meaningful associations between individual characteristics, such as religious beliefs and political orientation, and willingness to invoke the conscience clause.

## Discussion

The principle of the conscience clause has been a source of public debate in Polish society for years. While most citizens oppose refusal of legal services such as prenatal testing (73%), contraception (55%), or abortion (52%), medical professionals hold more varied views (49). Studies show that 39–53% of physicians and 27% of nurses consider invoking the conscience clause acceptable not only for abortion but also in contexts such as transplantation and palliative care (4).

The present study reflects these broader tendencies. It confirms that 90.3% of respondents support respecting patients’ beliefs, and 68.4% affirm the need to act according to their own conscience without compromising their moral values. Although 62.3% of patients (50) and 48.7% of nursing students (51) did not see religion as affecting care, Bülow et al. reported conflicts among staff with differing beliefs, especially in intensive care (52). Nurses were more likely than physicians to act against patients’ wishes in end-of-life situations, while 15–30% of religious physicians also failed to honor competent patients’ requests to discontinue therapy (53, 54).

Our findings further indicate that while 53.9% of respondents believe that personal views on death may justify invoking the conscience clause, and 46.5% accept its use in cases of organ retrieval, the most divergent opinions (33.9% vs. 37.2%) concerned disconnection of a brain-dead patient. For young nurses and midwives, this represents a particularly significant ethical dilemma. Previous studies show that professionals with stronger religious beliefs are more likely to continue life-sustaining treatment and less likely to forgo end-of-life therapy (53, 55–57) and greater attention to patients’ spiritual needs (58). In bioethical discourse, treatment withdrawal is sometimes equated with passive euthanasia (59–61), leading many religious professionals, particularly Catholics, to view it as a morally impermissible form of life-shortening. Studies by Guzowski et al. link high religiosity with lower acceptance of treatment withdrawal and stronger opposition to euthanasia, even in terminal cases (62), while findings by Musgrave and Soudry confirm similar attitudes among nurses and midwives (63). Religiosity is associated with views on invoking the conscience clause: religious professionals tend to be more likely to refuse participation in controversial procedures and less likely to refer patients elsewhere (64). Pew data indicate strong support for the conscience clause among conservative Catholics (55%), Protestants (68%), and Republicans (73%) (65). Both Polish and international research suggest that the clause is often perceived not only as an individual safeguard but also as a political or religious instrument, with religious and conservative beliefs reinforcing moral refusal, while liberal views emphasize patient autonomy and access to care (34, 64–67). This confirms international findings on the role of religiosity in shaping attitudes. In Poland, however, this influence is particularly pronounced, as the moral teaching of the Catholic Church remains strong and is often entangled in legal debates, thereby amplifying these effects compared with more secular contexts.

The odds ratio values observed in our models, although modest in explanatory power, provide meaningful insights into professional practice. For instance, an OR of 2.076 for religious versus nonreligious respondents (Table 6) indicates that religious professionals were approximately twice as likely to support the invocation of the conscience clause in the context of defining human death. This highlights the role of belief systems in ethically sensitive decisions and the need to consider individual values when developing guidelines and educational strategies. At the same time, it is important to note that some other statistically significant odds ratios were closer to 1, suggesting only small differences between groups. While these effects should not be dismissed, they should be interpreted with caution to avoid overstating their practical significance. Instead, such findings are best understood as pointing to subtle tendencies rather than strong predictive factors, complementing the more robust associations observed in the study.

Professional background also plays a role in shaping end-of-life decisions. While 87% of physicians prefer to decide individually, 70–78% of nurses support shared decision-making with the patient’s family (68, 69). This collaborative approach improves communication, reduces conflict, and lowers the likelihood of futile treatment. Nurses’ preferences may reflect their emphasis on beneficence and the value placed on family bonds (70–72).

Compared with abortion, ex mortuo transplantation generates less public controversy. Nevertheless, about one-third of nurses still report moral dilemmas surrounding it (2). Concerns include how brain death is defined, the possibility of misdiagnosis, and whether maintaining organ function may compromise a dignified death or bodily integrity (14, 73–76). Ethical discomfort also stems from perfusion techniques that temporarily restore circulation to evaluate or improve organs before transplantation (77). Taken together, these issues highlight the continuing need for clear ethical and legal guidelines on the use of the conscience clause in cases involving brain death.

Another important result of this study is the widespread support (81.8%) for developing consistent medical regulations on the conscience clause. Respondents emphasized the need for rules that protect both professional conscience and patient rights. Additionally, 45.3% of respondents believe that healthcare workers who invoke the clause should be required to refer the patient to another provider. At the same time, 53.9% of respondents oppose asking job candidates about their moral views on the conscience clause during the hiring process. Standardization of legal frameworks is also supported by Shaw et al., who argue that allowing conscientious objection may reduce covert resistance to organ donation and help align care with the staff’s beliefs by assigning alternative personnel (77). Card similarly argues that professionals who invoke the clause should be required to justify their decision, which should be subject to review (78).

This perspective is consistent with findings by Toro Flores et al. They note that although only 8.8% of nurses saw organ retrieval as a situation requiring the conscience clause, and only 5.9% pointed to the withdrawal of life-sustaining therapy, both contexts require deeper ethical, legal, and clinical reflection (79). The authors stress that healthcare workers may refuse participation in such procedures, provided that care is transferred to another provider, as abandonment or delays are ethically unacceptable (79). Other studies reinforce this view. In Poland, 67% of physicians and 39% of nurses support the obligation to refer patients (4). In South Korea, 68.7% of nurses prioritize patient rights over personal beliefs, while in Greece, the absence of referral policies discouraged professionals from invoking the clause (80, 81). These cross-national differences suggest that while concern for patient rights is widely shared, the balance between individual conscience and institutional safeguards varies across systems. In Poland, the relatively weaker referral framework may exacerbate tensions compared with contexts where clear referral obligations are in place.

The study also shows that fears of negative consequences are a major deterrent. Delays or denial of access to care are among the primary reasons why professionals may hesitate to invoke the conscience clause. In addition, participants expressed anxiety about social and professional repercussions, including loss of trust, damaged reputation, and professional backlash. Specifically, 56.9% feared legal action from patients’ families, 47.5% were concerned about a lack of peer support, and 45.7% worried about gossip or judgment from others. Others feared job loss (43.9%) or disciplinary action (41.6%). Similar concerns have been documented elsewhere. Voultsos et al. describe nurses’ fear of gossip, isolation, and workplace hostility when invoking the conscience clause (81). A UK study found that fear of losing one’s job discouraged professionals from expressing objections (82). Maxwell et al. reported similar concerns among pharmacists (83). Taken together, these findings raise questions about the lack of institutional protections, the absence of clear procedures, and insufficient training for managers, all of which contribute to inconsistent handling of conscience-based objections. In this context, our data confirm international reports of fear-driven reluctance, though the intensity of these concerns in Poland may reflect the combined effect of legal ambiguity and cultural pressures, including the dominant role of religion in shaping professional norms.

Our findings, therefore, align with international research showing that, although healthcare professionals often wish to act in harmony with their conscience, they rarely exercise this right in practice because of fear of conflict or negative repercussions (84, 85). For example, previous Polish studies conducted among physicians, nurses, and pharmacists demonstrated that many were concerned that invoking the right to the conscience clause could provoke conflicts with fellow healthcare professionals and patients. Moreover, pharmacists indicated that if the law were to allow them to invoke the conscience clause, it could limit patients’ freedom of choice (4, 34). Similarly, our results show that many students recognized significant ethical and legal conflicts, particularly in relation to family objections to organ donation, the absence of legal consequences for patients’ declarations of will, and the management of pregnant brain-dead patients (86). Studies from Poland and Slovakia likewise demonstrate that most nurses and pharmacists report moral conflict at work, but only a small minority have ever invoked the conscience clause (85, 87, 88). Concerns that conscience clause use might restrict patient access to legal care have also been highlighted by pharmacy students and pharmacists (89), echoing arguments by Montgomery (90) and Dickens (91) that patients’ rights to health and non-discriminatory access to services must remain a priority. Studies from Canada and elsewhere indicate that reluctance to use the conscience clause may also reflect a lack of institutional support or psychological burdens, not only religious convictions (85, 88, 92–96). This contrast suggests that while Polish findings share commonalities with international trends, the particularly strong role of cultural and legal contexts, especially the influence of Catholic doctrine and incomplete institutional safeguards, helps explain why some dilemmas appear more acute in Poland than in more secular or procedurally robust systems.

Another conclusion that emerges is that while the majority of respondents do not support abolishing the conscience clause, they strongly favour more precise regulations, especially concerning professional duties. Nearly 80% believe that medical experts should develop such rules. Other studies support this finding: 75% of physicians, 44% of nurses, and 59% of pharmacists consider the current framework vague, and more than 75% support clearer criteria (4). Both religious and religiously ambivalent professionals point to the risk of misuse stemming from legal ambiguity (34).

Ultimately, this study highlights the impact of political orientation on attitudes toward organ donation. Centrists, more than liberals or conservatives, supported the right to refuse participation in *ex mortuo* transplantation. Political orientation, along with age and education, was associated with positions on post-mortem donation. Prior research shows higher support among younger, highly educated, and liberal respondents. At the same time, conservatives were more likely to express moral or ethical objections, often linked to tradition, religious values, or distrust in public healthcare (97). Given the emotional weight of transplantation, young nurses and midwives, who are still forming their professional identity and often lacking clinical experience, frequently view ex mortuo donation as a profound ethical dilemma.

## Limitations

This study has several limitations that should be taken into account when interpreting the results. Firstly, the research was conducted among master’s students of nursing and midwifery from a single Polish medical university, which gives it a local character and limits the generalizability of the findings to a broader student population in Poznan or Poland. As a result, caution is needed when applying these findings to other contexts, and future research should include multi-institutional studies to enhance generalizability. Secondly, although the response rate was high (91.8%), the sample remains relatively small and does not include the views of the 24 students who declined to participate. This limitation reduces the statistical power of the study, and future research should seek to include larger and more representative samples. Thirdly, the study group was heavily gender-skewed, with female students vastly outnumbering males. However, this reflects the broader gender distribution within nursing and midwifery programs in Poland. This imbalance may restrict the expression of more varied viewpoints, and future research should aim to recruit more diverse and multi-institutional samples and consider strategies to mitigate gender bias to enhance the representativeness of findings. Fourthly, future research should aim to include students from other faculties, such as medicine, psychology, or emergency medicine, who also encounter patients in end-of-life situations. Broadening the scope in this way would provide a more comprehensive view of attitudes across different healthcare disciplines. Fifthly, although the questionnaire was reviewed by domain experts and piloted, it was not formally validated, which may impact the reliability of certain constructs. This limitation means that some degree of measurement bias cannot be excluded; therefore, future studies should incorporate formal validation processes to strengthen the robustness and comparability of findings. Sixth, the study relied solely on self-reported, declarative data, which may not accurately reflect actual clinical behaviors or ethical decision-making under pressure. This restriction may limit the ecological validity of the results, and future research should employ qualitative or observational methodologies to better capture real-life practices and decision-making processes. In particular, using in-depth interviews, focus groups, or ethnographic observation could be especially valuable in uncovering the nuanced reasoning behind these attitudes. Seventh, the cross-sectional design captures a static view of students’ opinions, which may evolve as their clinical experience increases. Thus, the findings may not reflect changes over time, and longitudinal studies are recommended to track how attitudes develop with clinical practice. Another limitation is the potential risk of social desirability bias due to in-class data collection conducted under the supervision of the researcher. This may have influenced participants to give more socially desirable responses, and future studies should use fully anonymous online surveys or similar methods to reduce researcher influence. A limitation of our study is the relatively low explanatory power of the logistic regression models (Nagelkerke R^2^ < 0.108), which is common in social science research but calls for cautious interpretation of the findings. Finally, the absence of a qualitative component limited our ability to explore the deeper reasoning behind the students’ attitudes. As a consequence, important nuances and complexities may have been overlooked, and future research should integrate qualitative methods to better capture these dimensions more comprehensively. Additionally, cultural and systemic factors typical for Polish society, i.e., the influence of moral teachings of the Catholic Church, the legal regulations of the conscience clause, and the particular structure of the healthcare system, may have influenced students’ perceptions of brain death and the conscience clause. Consequently, these findings cannot be generalized to other countries, and in order to make international comparisons, future studies should also consider the local context.

Despite these limitations, the study offers several strengths. Most importantly, it is one of the first to investigate the conscience clause in the context of brain death among Polish master’s students in nursing and midwifery, thereby making a distinctive contribution to the literature. It provides preliminary data on how young healthcare professionals perceive ethical and legal dilemmas surrounding brain death. These findings may inform future educational efforts, support policy discussions on the conscience clause, and stimulate broader interdisciplinary research on death, dying, and professional responsibility in healthcare.

## Conclusion

This study highlights the complexity of ethical and legal dilemmas faced by young nurses and midwives in the context of brain death and the conscience clause. While most respondents support respecting patient beliefs and acting in line with their own conscience, many expressed concern about the social, legal, and professional consequences of conscientious objection. The findings also show that religiosity and political orientation are associated with views on brain death and organ donation. Respondents clearly preferred structured, expert-led regulation over hospital-level discretion or the abolition of the clause.

Given the preliminary and local nature of these findings, the conclusions should be interpreted with caution. Nevertheless, they point to several areas where policy and educational efforts could be considered in order to support ethically grounded clinical practice.

1. National guidelines on the conscience clause in brain death-related care could be developed through collaboration between interdisciplinary expert panels (including ethicists, legal scholars, clinicians, and representatives of professional bodies) and government agencies.
2. Legal safeguards may be needed to protect healthcare professionals from discrimination, while also ensuring continuity of patient care. These could include clear procedures for documenting conscientious objection and structured referral mechanisms.
3. Ethics training could be more explicitly integrated into nursing and midwifery curricula, for example, through case studies, simulation exercises, and interdisciplinary teaching that build both reflective and practical decision-making skills.
4. Future research, particularly qualitative and longitudinal studies, will be crucial to deepen understanding of how clinical experience shapes ethical decision-making and to test whether the suggested measures are feasible and effective.

At the same time, while making these recommendations, future studies and policy work should also address potential barriers to implementation, including institutional resistance, limited resources, regional policy differences, and the influence of religion.

## Data Availability

The original contributions presented in the study are included in the article/supplementary material, further inquiries can be directed to the corresponding author.
